# Supplementary material for: Pregnant Women Infected with Zika Virus Show Higher Viral Load and Immunoregulatory Cytokines Profile with CXCL10 Increase
Source: Viruses. 2021 Jan 8;13(1):80. doi: 10.3390/v13010080 (PMC7827657; doi:10.3390/v13010080)
Supplement: Supplementary file 1 [file viruses-13-00080-s001.pdf]

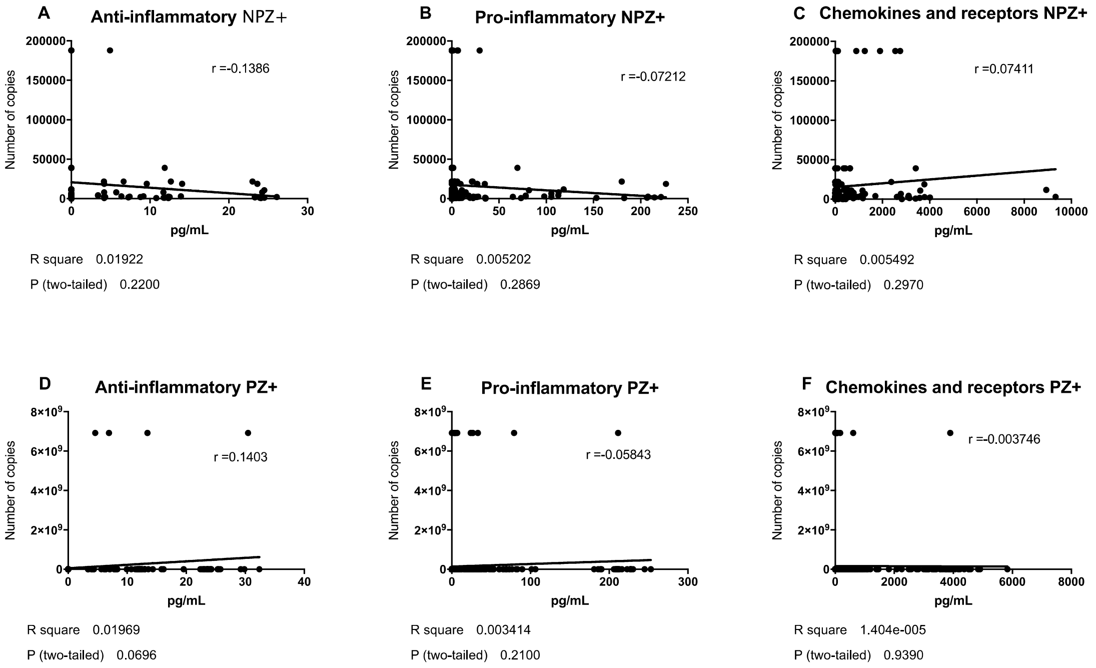

Supplementary Figure S1. Relationship between viral load and serum cytokine concentration between NPZ+ and PZ+ infected groups. NPZ+ (A) anti-inflammatory cytokines, (B) proinflammatory, (C) chemokines. PZ+ (D) anti-inflammatory cytokines, (E) proinflammatory (F) chemokines and receptors. Correlation analysis of Pearson's r test.

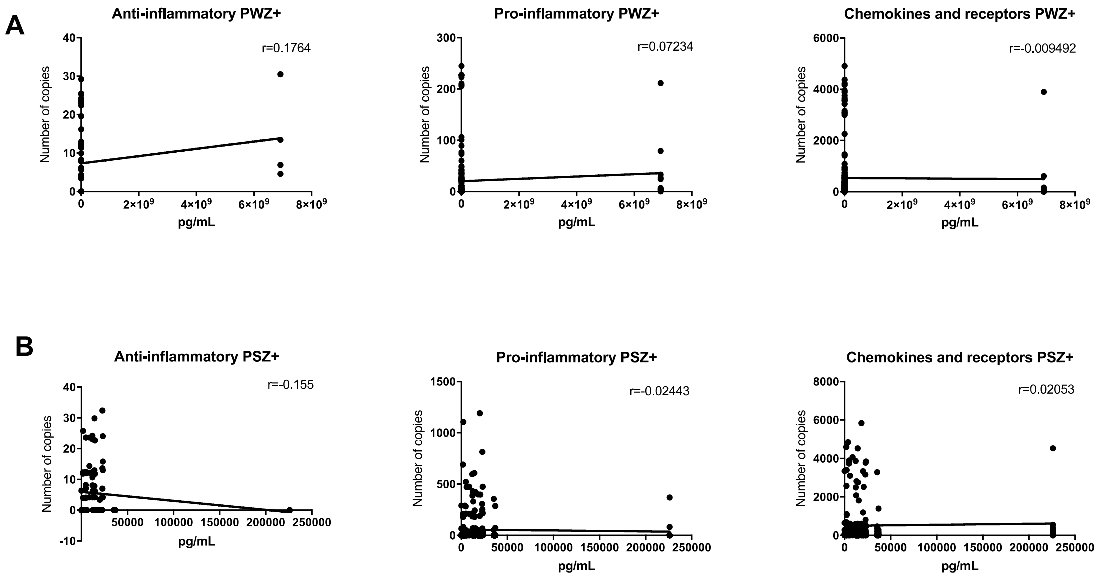

Supplementary Figure S2. Relationship between viral load and serum cytokine concentration between infected groups. *(A)* PWZ+ and *(B)* PSZ+. *A*nti-inflammatory cytokines, proinflammatory, chemokines and receptors. Correlation analysis of Pearson's r test. Correlation analysis of Pearson's r test.

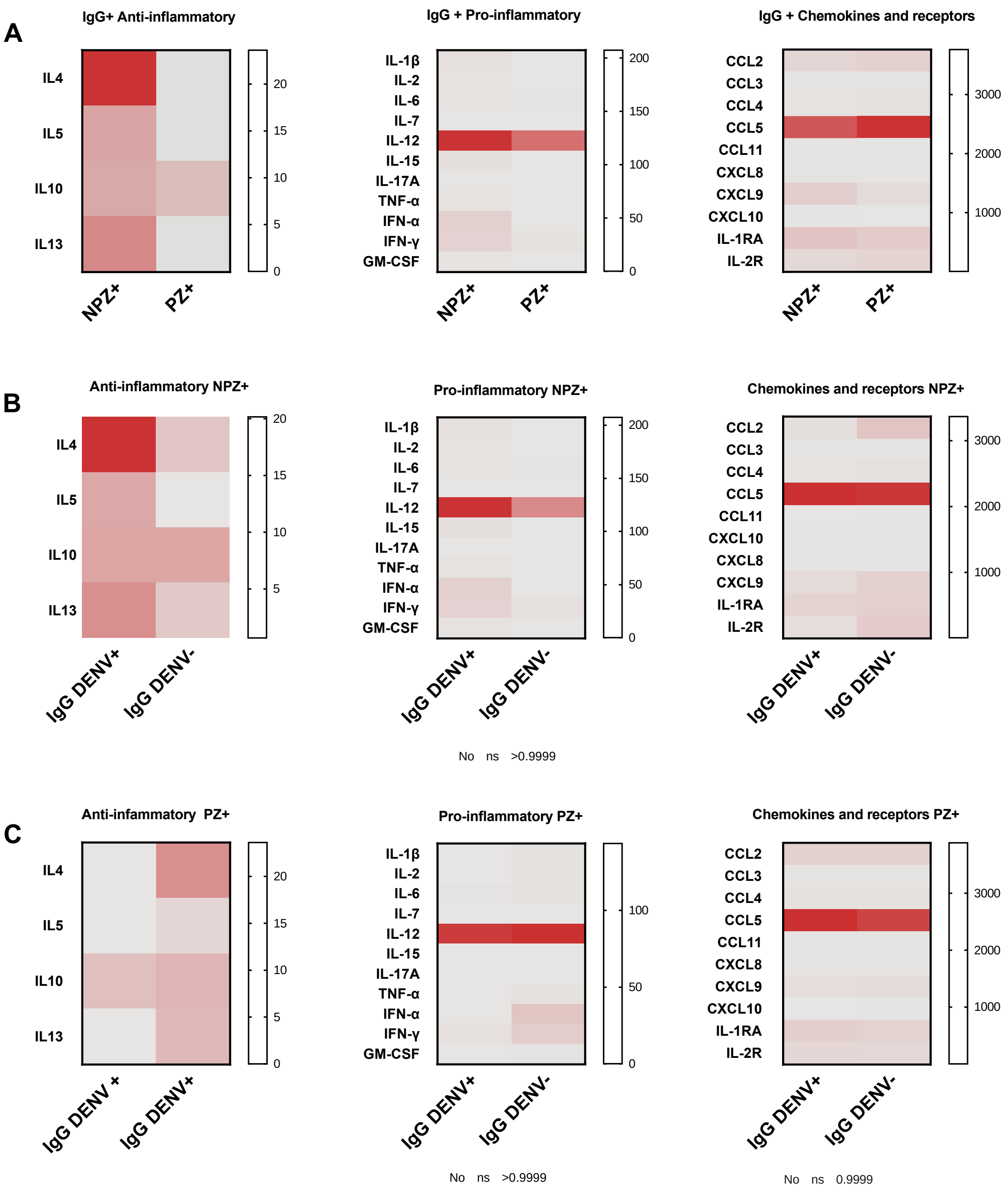

Supplementary Figure S3. Relationship between IgG DENV infected groups. (A) Relationship between IgG DENV NPZ+ and PZ+ infected groups, (B) NPZ+ positive vs negative IgG DENV (C) PZ+ positive vs negative IgG DENV. Sidak's multiple comparisons test p>0.005.

Supplementary Table S1. Cytokine concentrations in nonpregnant healthy women and nonpregnant women infected with ZIKV. Healthy nonpregnant women (NPH=14); Nonpregnant women ZIKV positives (NPZ+=22); Cytokines were determined by a Luminex 25-plex assay (pg/mL) and grouped in Anti-inflammatory, proinflammatory and chemokines and receptors. NS: not statistically significant; BDL: below detection limit

Experimental group								
	NPH			NPZ+			*P*	
Cytokines	Median.	Q1	Q3	Median	Q1	Q3		
Anti-inflammatory								
IL-4	BDL	BDL	BDL	BDL	BDL	23.31	0.0554	NS
IL-5	BDL	BDL	BDL	BDL	BDL	4.17	0.0107	*
IL-10	BDL	BDL	BDL	6.945	3.4	9.57	<0.0001	****
IL-13	BDL	BDL	BDL	BDL	BDL	12.21	0.0051	**
Pro-inflammatory								
IL-1 β	0.27	BDL	0.27	0.16	BDL	6.71	0.3527	NS
IL-2	BDL	BDL	BDL	0.7701	BDL	3.28	0.0418	*
IL-6	BDL	BDL	BDL	2.004	0.91	3.45	0.0026	**
IL-7	BDL	BDL	BDL	BDL	BDL	BDL	0.2925	NS
IL-12	80.02	71.91	100.2	112.9	79.15	200.67	0.0422	*
IL-15	BDL	BDL	BDL	BDL	BDL	8.16	0.0554	NS
IL-17 A	BDL	BDL	BDL	BDL	BDL	BDL	0.2667	NS
TNF-α	BDL	BDL	BDL	BDL	BDL	2.71	0.0105	*
IFN-α	BDL	BDL	BDL	10.8	BDL	25.28	0.0819	NS
IFN-γ	BDL	BDL	BDL	8.07	1.87	22.17	<0.0001	****
GM-CSF	BDL	BDL	BDL	BDL	BDL	3.2	0.0107	*
Chemokines								
CCL2	323.4	244.17	452.06	478.2	234.17	879.04	0.3471	NS
CCL3	BDL	BDL	BDL	12.39	2.36	24.34	0.006	**
CCL4	125.6	104.29	133.62	103.3	54.34	117.55	0.0357	*
CCL5	2825	2397.46	3486.13	3136	2654.09	3598.16	0.4654	NS
CCL11	27.71	19.45	40.37	8.48	4.87	26.71	0.0095	**
CXCL8	BDL	BDL	BDL	19.24	6.19	38.31	<0.0001	****
CXCL9	48.59	41.55	148.87	356.1	111.66	578.61	0.0002	***
CXCL10	7.9	6.61	11.4	9.519	5.65	12.87	0.7067	NS
Receptors								
IL-1RA	233.8	158.9	294.61	400.4	291.84	631.8	0.0005	***
IL-2R	241.2	209.2	305.26	421.4	148.96	565.73	0.1726	NS

Supplementary Table S2. Cytokines in nonpregnant and pregnant women not infected with ZIKV. Cytokines were determined by a Luminex 25-plex assay. Groups: Healthy nonpregnant women (NPH=14); Healthy pregnant women (PH=30). Cytokines were Cytokines were determined by a Luminex 25-plex assay (pg/mL) and grouped in Anti-inflammatory, proinflammatory and chemokines and receptors. NS: not statistically significant; BDL: below detection limit.

Experimental group								
	NPH			PH			*P*	
Cytokines	Median	Q1	Q3	Median	Q1	Q3		
Anti-inflammatory								
IL-4	BDL	BDL	BDL	BDL	BDL	22.7	0.1218	NS
IL-5	BDL	BDL	BDL	BDL	BDL	4.02	0.0199	*
IL-10	BDL	BDL	BDL	BDL	BDL	5.55	0.02	*
IL-13	BDL	BDL	BDL	BDL	BDL	11.66	0.136	NS
Pro-inflammatory								
IL-1β	0.27	BDL	0.27	BDL	BDL	4.02	0.6904	NS
IL-2	BDL	BDL	BDL	BDL	BDL	11.66	<0.0001	****
IL-6	BDL	BDL	BDL	BDL	BDL	5.55	<0.0001	****
IL-7	BDL	BDL	BDL	BDL	BDL	21.71	>0.999	NS
IL-12	80.02	71.91	100.2	78.27	52.36	22.7	0.9652	NS
IL-15	BDL	BDL	BDL	BDL	BDL	2.13	0.2326	NS
IL-17 A	BDL	BDL	BDL	BDL	BDL	2.35	0.556	NS
TNF-α	BDL	BDL	BDL	BDL	BDL	3	0.02	*
IFN-α	BDL	BDL	BDL	BDL	BDL	5.15	0.5618	NS
IFN-γ	BDL	BDL	BDL	BDL	BDL	191.38	0.0097	**
GM-CSF	BDL	BDL	BDL	BDL	BDL	6.18	0.0199	*
Chemokines								
CCL2	323.4	244.17	452.06	258.7	165.93	329.41	0.0625	NS
CCL3	BDL	BDL	BDL	6.76	BDL	19.28	0.0304	*
CCL4	125.6	104.29	133.62	88.77	64.28	121.59	0.0155	*
CCL5	2825	2397.46	3486.13	3498	3177.52	3890.53	0.0448	*
CCL11	27.71	19.45	40.37	8.91	7.31	13.34	<0.0001	****
CXCL8	BDL	BDL	BDL	2.62	BDL	10.5	0.0017	**
CXCL9	48.59	41.55	148.87	62.03	37.68	127.49	0.995	NS
CXCL10	7.9	6.61	11.4	1.21	0.9	1.73	<0.0001	****
Receptors								
IL-1RA	233.8	158.9	294.61	204.4	137.5	298.01	0.4811	NS
IL-2R	241.2	209.2	305.26	206.5	103.58	307.7	0.2319	NS

Supplementary Table S3. Cytokines in healthy pregnant women and pregnant women infected with ZIKV., healthy pregnant women (PH= 30), Pregnant women ZIKV positive (PZ+= 44). Cytokines were determined by a Luminex 25-plex assay (pg/mL) and grouped in Anti-inflammatory, proinflammatory and chemokines and receptors. NS: not statistically significant; BDL: below detection limit.

Experimental group								
	PH			PZ+			*P*	
Cytokines	Median	Q1	Q2	Median	Q1	Q3		
Anti-inflammatory								
IL-4	BDL	BDL	22.7	BDL	BDL	23.96	0.0523	NS
IL-5	BDL	BDL	4.02	BDL	BDL	4.16	0.2217	NS
IL-10	BDL	BDL	5.55	6.15	BDL	7.91	0.0009	***
IL-13	BDL	BDL	11.66	BDL	BDL	12.05	0.0995	NS
Pro-inflammatory								
IL-1**β**	BDL	BDL	4.02	1.214	BDL	7.22	0.251	NS
IL-2	BDL	BDL	11.66	1.31	BDL	3.59	0.2154	NS
IL-6	BDL	BDL	5.55	2.69	BDL	4.06	0.0073	**
IL-7	BDL	BDL	21.71	BDL	BDL	BDL	0.1418	NS
IL-12	78.27	52.36	22.7	141.35	65.66	211.47	0.0804	NS
IL-15	BDL	BDL	2.13	BDL	BDL	17.36	0.2145	NS
IL-17 A	BDL	BDL	2.35	BDL	BDL	0.06	0.0411	*
TNF-**α**	BDL	BDL	3.00	1.17	BDL	2.85	0.0484	*
IFN-**α**	BDL	BDL	5.15	23.41	BDL	34.36	0.0022	**
IFN-**γ**	BDL	BDL	191.38	12.12	BDL	22.87	0.0454	*
GM-CSF	BDL	BDL	6.18	BDL	BDL	3.22	0.0675	NS
Chemokines								
CCL2	258.7	165.93	329.41	421.9	212.92	604.85	0.0016	**
CCL3	6.76	BDL	19.28	14.53	2.36	31	0.0853	NS
CCL4	88.77	64.28	121.59	75	54.43	101.19	0.1998	NS
CCL5	3498	3177.52	3890.53	3568	3049.29	3968.19	0.9346	NS
CCL11	8.91	7.31	13.34	7.159	4.82	14.29	0.0695	NS
CXCL8	2.62	BDL	10.5	9.836	1.78	17.58	0.0123	*
CXCL9	62.03	37.68	127.49	166.7	82.1	262.35	<0.0001	****
CXCL10	1.21	0.9	1.73	6.68	3.24	10.35	<0.0001	****
Receptors								
IL-1RA	204.4	137.5	298.01	411.6	286.58	609.49	<BDL01	****
IL-2R	206.5	103.58	307.7	264.6	142.36	531.52	0.0319	*

Supplementary Table S4. Cytokines in nonpregnant and pregnant women infected with ZIKV. Nonpregnant women with ZIKV infection (NPZ+=22); Pregnant women with ZIKV infection (PZ+=44) Cytokines were Cytokines were determined by a Luminex 25-plex assay (pg/mL) and grouped in Anti-inflammatory, proinflammatory and chemokines and receptors. NS: not statistically significant; BDL: below detection limit.

	Experimental group							
	NPZ+			PZ+			*P*	
Cytokines	Median	Q1	Q3	Median	Q1	Q3		
Anti-inflammatory								
IL-4	BDL	BDL	23.31	BDL	BDL	23.96	0.4101	NS
IL-5	BDL	BDL	4.17	BDL	BDL	4.16	0.6445	NS
IL-10	6.95	3.4	9.57	6.15	BDL	7.91	0.1573	NS
IL-13	BDL	BDL	12.21	BDL	BDL	12.05	0.9446	NS
Pro-inflammatory								
IL-1 **β**	0.14	BDL	6.71	1.21	BDL	7.22	0.6383	NS
IL-2	0.77	BDL	3.28	1.31	BDL	3.59	0.7047	NS
IL-6	2	0.91	3.45	2.69	BDL	4.06	0.9155	NS
IL-7	BDL	BDL	BDL	BDL	BDL	BDL	0.2925	NS
IL-12	112.9	79.15	200.67	141.35	65.67	211.47	0.9435	NS
IL-15	BDL	BDL	8.16	BDL	BDL	17.36	0.8997	NS
IL-17 A	BDL	BDL	BDL	BDL	BDL	0.06	0.2891	NS
TNF-**α**	BDL	BDL	2.71	1.17	BDL	2.85	0.34588	NS
IFN-**α**	10.8	BDL	25.28	23.41	BDL	34.36	0.3546	NS
IFN-**γ**	8.069	1.87	22.17	12.12	BDL	22.87	0.7158	NS
GM-CSF	BDL	BDL	3.2	BDL	BDL	3.22	0.3771	NS
Chemokines								
CCL2	478.2	234.17	879.04	421.9	212.93	604.85	0.48446	NS
CCL3	12.39	2.36	24.34	14.53	2.36	31	0.7102	NS
CCL4	103.3	54.34	117.55	75	54.43	101.19	0.3961	NS
CCL5	3136	2654.09	3598.16	3568	3049.29	3968.19	0.1266	NS
CCL11	8.476	4.87	26.71	7.16	4.82	14.29	0.2015	NS
CXCL8	19.24	6.19	38.31	9.84	1.78	17.58	0.058	NS
CXCL9	356.1	111.66	578.61	166.7	82.1	262.35	0.027	*
CXCL10	9.52	5.65	12.87	6.68	3.24	10.35	0.0959	NS
Receptors								
IL-1RA	400.4	291.84	631.8	411.6	286.58	609.49	0.9973	NS
IL-2R	421.4	148.96	565.73	264.6	142.36	531.52	0.5102	NS

Supplementary Table S5. Cytokines in healthy pregnant women and pregnant women infected with ZIKV by trimester. Healthy pregnant women (PH; 1st=10, 2nd=15 and 3rd=5), pregnant women infected with ZIKV (PZ+; 1st=11, 2nd=27 and 3rd=6). Cytokines were determined by a Luminex 25-plex assay (pg/mL) and grouped in Anti-inflammatory, proinflammatory and chemokines and receptors. NS: not statistically significant; BDL: below detection limit.

	1st trimester								2nd trimester								3rd trimester							
	PH			PZ+			*P*		PH			PZ+			*P*		PH			PZ+			p	
Cytokines	Median	Q1	Q3	Median	Q1	Q3			Median	Q1	Q3	Median	Q1	Q3			Median	Q1	Q3	Median	Q1	Q3		
Anti-inflammatory																								
IL-4	BDL	BDL	17.02	23.00	BDL	24.62	0.01	*	BDL	BDL	11.20	BDL	BDL	23.62	>0.99	NS	22.70	BDL	23.31	23.00	11.35	24.70	>0.99	NS
IL-5	BDL	BDL	2.97	4.03	BDL	4.14	0.02	*	BDL	BDL	1.98	BDL	BDL	4.15	>0.99	NS	4.03	BDL	4.44	4.03	1.98	4.2	>0.99	NS
IL-10	BDL	BDL	4.18	7.80	1.70	8.15	0.02	*	BDL	BDL	2.73	1.70	BDL	6.79	0.03	*	5.46	BDL	5.79	7.02	4.78	8.13	>0.99	NS
IL-13	BDL	BDL	8.65	11.70	BDL	12.09	0.01	*	BDL	BDL	8.74	BDL	BDL	11.85	>0.99	NS	11.85	BDL	11.85	12.33	5.93	12.49	>0.99	NS
Pro-inflammatory																								
IL-1 β	0.14	BDL	7.32	4.32	BDL	7.57	>0.99	NS	BDL	BDL	1.97	BDL	BDL	6.52	0.30	NS	3.81	BDL	6.76	6.76	2.41	11.93	>0.99	NS
IL-2	BDL	BDL	3.14	3.18	BDL	3.25	0.28	NS	BDL	BDL	2.62	BDL	BDL	3.59	>0.99	NS	2.90	1.54	3.18	3.04	1.45	3.73	>0.99	NS
IL-6	0.90	0.91	2.23	2.55	BDL	3.44	0.99	NS	BDL	BDL	0.66	2.55	BDL	5.32	>0.99	NS	2.13	BDL	2.13	3.11	0.96	3.83	>0.99	NS
IL-7	BDL	BDL	BDL	BDL	BDL	0.00	>0.99	NS	BDL	BDL	BDL	BDL	BDL	BDL	>0.99	NS	BDL	BDL	BDL	BDL	BDL	BDL	>0.99	NS
IL-12	73.90	51.47	179.29	205.90	83.00	226.95	>0.99	NS	62.27	51.65	140.54	77.39	61.59	211.22	>0.99	NS	205.23	124.90	218.74	188.77	183.37	209.11	0.22	NS
IL-15	BDL	BDL	10.47	BDL	BDL	6.49	0.31	NS	BDL	BDL	BDL	BDL	BDL	18.72	>0.99	NS	BDL	BDL	10.64	8.24	BDL	31.96	>0.99	NS
IL-17 A	BDL	BDL	BDL	BDL	BDL	0.00	0.16	NS	BDL	BDL	BDL	BDL	BDL	BDL	0.21	NS	BDL	BDL	BDL	0.27	BDL	0.73	>0.99	NS
TNF-α	BDL	BDL	1.65	2.42	BDL	3.04	0.99	NS	BDL	BDL	1.17	BDL	BDL	2.64	0.28	NS	2.42	BDL	2.49	2.79	1.25	3.69	>0.99	NS
IFN-α	BDL	BDL	14.80	28.60	BDL	37.92	0.28	NS	BDL	BDL	9.19	10.80	BDL	32.80	>0.99	NS	25.52	BDL	26.53	27.92	10.81	33.35	>0.99	NS
IFN-γ	0.69	BDL	16.62	20.70	1.67	22.43	>0.99	NS	BDL	BDL	8.70	3.35	BDL	22.77	>0.99	NS	21.71	1.37	23.23	19.95	10.18	21.87	0.48	NS
GM-CSF	BDL	BDL	2.36	3.19	BDL	3.23	>0.99	NS	BDL	BDL	1.57	BDL	BDL	3.22	>0.99	NS	3.18	BDL	3.20	3.20	1.59	3.23	>0.99	NS
Chemokines																								
CCL2	245.08	116.90	427.01	482.85	264.85	608.42	>0.99	NS	285.51	193.20	327.42	450.88	230.20	643.81	>0.99	NS	203.13	157.67	315.43	323.09	254.56	408.23	>0.99	NS
CCL3	8.95	BDL	19.28	12.53	BDL	25.08	>0.99	NS	BDL	BDL	19.28	19.28	8.81	32.45	0.002	**	7.63	4.11	9.44	8.46	2.06	13.91	>0.99	NS
CCL4	84.57	64.28	124.58	67.54	37.96	104.08	>0.99	NS	101.22	72.08	127.61	80.53	54.61	109.40	>0.99	NS	73.72	72.43	88.77	73.08	46.97	87.75	>0.99	NS
CCL5	3576.99	3276.94	4414.62	3649.34	3291.87	4056.57	>0.99	NS	3483.02	3118.88	3775.04	3490.23	2859.61	4000.65	>0.99	NS	3773.79	3395.06	4719.03	3338.14	3019.30	3756.30	>0.99	NS
CCL11	12.48	7.65	16.27	13.58	8.77	15.33	>0.99	NS	8.48	7.01	13.28	5.78	3.39	10.99	>0.99	NS	8.67	8.48	12.93	5.47	4.99	13.13	>0.99	NS
CXCL8	66.15	55.40	139.24	249.83	100.21	299.85	>0.99	NS	62.03	42.27	96.02	166.73	113.50	230.56	0.002	**	36.39	28.30	110.67	82.46	74.59	181.32	>0.99	NS
CXCL9	6.28	BDL	11.81	6.07	BDL	11.99	099	NS	BDL	0.00	7.71	9.84	2.09	21.16	0.99	NS	11.04	8.04	13.28	13.44	9.07	16.47	>0.99	NS
CXCL10	1.32	0.54	1.63	14.29	6.58	22.24	0.99	NS	1.13	0.89	1.68	5.95	2.84	8.95	0.99	NS	1.29	1.27	2.08	6.07	3.88	7.46	>0.99	NS
Receptors																								
IL-1RA	169.82	121.49	204.77	284.24	204.17	575.50	>0.99	NS	219.26	181.13	317.99	426.68	313.18	591.97	>0.99	NS	261.41	190.83	300.87	387.99	318.87	752.25	>0.99	NS
IL-2R	182.45	98.45	269.00	250.64	147.55	434.42	>0.99	NS	217.31	137.36	293.37	336.51	159.12	557.22	>0.99	NS	133.05	108.24	356.30	161.11	113.25	321.94	>0.99	NS

Supplementary Table S6. Cytokines in weakly symptomatic pregnant and symptomatic pregnant women infected with ZIKV. Weakly symptomatic pregnant women (PWZ+= 19) and symptomatic pregnant women (PSZ+= 25). Cytokines were Cytokines were determined by a Luminex 25-plex assay (pg/mL) and grouped in Anti-inflammatory, proinflammatory and chemokines and receptors. NS: not statistically significant; BDL: below detection limit.

Experimental group								
	PWZ+			PSZ+			*P*	
Cytokines	Median	Q1	Q3	Median	Q1	Q3		
Anti-inflammatory								
IL-4	23.00	BDL	24.78	0.00	BDL	23.62	0.240	NS
IL-5	4.03	BDL	4.17	0.00	BDL	4.17	0.2737	NS
IL-10	6.17	1.70	7.93	3.40	BDL	7.87	0.3996	NS
IL-13	11.70	BDL	12.01	0.00	BDL	12.17	0.6340	NS
Pro-inflammatory								
IL-1 **β**	4.82	0.00	8.72	0.00	0.00	6.76	0.3661	NS
IL-2	3.18	0.00	4.01	0.00	0.00	3.59	0.1555	NS
IL-6	2.82	0.00	3.74	1.93	0.00	5.27	0.8521	NS
IL-7	0.00	0.00	0.00	0.00	0.00	0.00	0.6221	NS
IL-12	208.27	83.00	226.94	81.75	60.45	208.44	0.0582	NS
IL-15	0.00	0.00	0.00	0.00	0.00	0.27	0.5336	NS
IL-17 A	0.00	0.00	29.65	0.00	0.00	15.28	0.3712	NS
TNF-**α**	2.49	0.00	3.04	0.00	0.00	2.79	0.1123	NS
IFN-**α**	28.60	10.80	36.18	0.00	0.00	32.13	0.0495	*
IFN-**γ**	20.70	0.00	22.91	3.35	0.00	20.75	0.4920	NS
GM-CSF	3.17	0.00	3.24	0.00	0.00	3.22	0.2708	NS
Chemokines								
CCL2	343.08	160.80	608.42	435.31	316.20	604.28	0.5071	NS
CCL3	12.77	0.00	22.88	15.57	5.38	31.95	<0.0001	****
CCL4	68.86	48.43	105.30	80.36	54.43	97.10	0.7030	NS
CCL5	3649.34	3122.89	3990.31	3343.12	2758.20	3899.02	0.4244	NS
CCL11	14.19	9.80	15.89	4.94	3.20	7.01	<0.0001	****
CXCL8	8.76	4.05	13.46	13.44	1.05	28.61	0.2892	NS
CXCL9	208.70	60.22	299.85	162.62	110.67	215.93	0.9392	NS
CXCL10	12.30	2.80	17.85	6.07	3.52	8.57	0.1566	NS
Receptors								
IL-1RA	492.17	239.27	622.47	399.81	307.37	521.37	0.7919	NS
IL-2R	250.64	147.55	487.98	307.03	142.77	544.97	0.9207	NS
